# Radiation-induced miR-208a increases the proliferation and radioresistance by targeting p21 in human lung cancer cells

**DOI:** 10.1186/s13046-016-0285-3

**Published:** 2016-01-12

**Authors:** Yiting Tang, Yayun Cui, Zengpeng Li, Zhuqing Jiao, Yong Zhang, Yan He, Guangxia Cheng, Qunyan Zhou, Wenjie Wang, Xifa Zhou, Judong Luo, Shuyu Zhang

**Affiliations:** Department of Radiation Oncology, Changzhou Cancer Hospital, Soochow University, Changzhou, 213001 China; Department of Radiation Oncology, Anhui Provincial Hospital, Hefei, 213001 China; State Key Laboratory Breeding Base of Marine Genetic Resources, Third Institute of Oceanography, State Oceanic Administration, Xiamen, 361005 China; Department School of Information Science and Engineering, Changzhou University, Changzhou, 213164 China; Department of Radiation Oncology, Shandong Cancer Hospital and Institute, Shandong University, Jinan, 250117 China; School of Radiation Medicine and Protection and Collaborative Innovation Center of Radiation Medicine of Jiangsu Higher Education Institutions, Soochow University, Suzhou, 215123 China; Department of Gastroenterology, First People’s Hospital of Xuzhou, Xuzhou, 221002 China; Department of Gastroenterology, Wuxi People’s Hospital Affiliated to Nanjing Medical University, Wuxi, 214002 China

**Keywords:** miR-208a, p21, Proliferation, Radioresistance, Serum exosomes, Lung cancer

## Abstract

**Background:**

Lung cancer has long been the most dangerous malignant tumor among males in both well developed and poorly developed countries. Radiotherapy plays a critical role in the curative management of inoperable non-small cell lung cancer (NSCLC) and is also used as a post-surgical treatment in lung cancer patients. Radioresistance is an important factor that limits the efficacy of radiotherapy for NSCLC patients. Increasing evidence suggests that microRNAs (miRNAs) possess diverse cellular regulatory roles in radiation responses.

**Methods:**

In this study, we used miRNA microarray technology to identify serum miRNAs that were differentially expressed before and after radiotherapy in lung cancer patients. We further examined the biological function of miR-208a on cell viability, apoptotic death and cell cycle distribution in human lung cancer cells and explored the probable mechanism.

**Results:**

Nine miRNAs, including miR-29b-3p, miR-200a-3p, and miR-126-3p were significantly down-regulated, whereas miR-208a was the only miRNA that was up-regulated in the serum of the patients after radiation treatment (*P* < 0.05). The expression of miR-208a could be induced by X-ray irradiation in lung cancer cells. Forced expression of miR-208a promoted cell proliferation and induced radioresistance via targeting p21 with a corresponding activation of the AKT/mTOR pathway in lung cancer cells, whereas down-regulation of miR-208a resulted in the opposite effects. In addition, down-regulation of miR-208a increased the percentage of cells undergoing apoptosis and inhibited the G1 phase arrest in NSCLC cells. Moreover, miR-208a from the serum exosome fraction of lung cancer patients could shuttle to A549 cells in a time-dependent manner, which was likely to contribute to the subsequent biological effects.

**Conclusions:**

The present study provides evidence that miR-208a can affect the proliferation and radiosensitivity of human lung cancer cells by targeting p21 and can be transported by exosomes. Thus, miR-208a may serve as a potential therapeutic target for lung cancer patients.

**Electronic supplementary material:**

The online version of this article (doi:10.1186/s13046-016-0285-3) contains supplementary material, which is available to authorized users.

## Background

Lung cancer has long been the most dangerous malignant tumor among males in both well developed and poorly developed countries and has surpassed breast cancer as the leading cause of cancer death among females in well developed countries [1]. Non-small cell lung cancer (NSCLC) and small cell lung cancer (SCLC) are the main types of lung cancer according to morphologic type, with NSCLC accounting for ~80–85 % of the tumors diagnosed in all lung cancer patients [2]. Radiotherapy plays a critical role in the curative management for inoperable NSCLC and shows promise as a post-surgical treatment for lung cancer patients [3]. One encouraging report suggested that stereotactic ablative radiotherapy (SABR) was a valuable adjunctive treatment for operable stage I NSCLC and that SABR might lead to better overall survival than surgery alone for operable clinical stage I NSCLC [4].

Radioresistance is an important factor that limits the efficacy of radiotherapy for NSCLC patients. Resistance to radiotherapy can be attributed either to intrinsic radioresistance of the tumor cells within the hypoxia microenvironment or to resistance acquired during fractionated radiotherapy [5, 6]. One of the molecular events by which tumors become radioresistant is radiation-induced activation of signal transduction pathways, such as those regulated by membrane-bound receptor tyrosine kinases (RTKs) [5]. Hypoxia-inducible factor 1α (HIF-1α) and reactive oxygen species (ROS) have been reported to mediate radiation-induced invasiveness in lung carcinoma cells [7]. However, the key molecules involved in radiation-induced radioresistance remain poorly understood. Thus, knowledge of the functional mechanisms is urgently warranted to overcome radioresistance during cancer therapy.

MicroRNAs (miRNAs) are small non-coding RNAs that specifically regulate gene expression by directly cleaving the targeted mRNAs or inhibiting translation by interacting with the 3′ untranslated regions (UTRs) of the mRNA targets to increase or inhibit their translation [8]. Increasing evidence suggests that miRNAs possess diverse cellular regulatory roles, and some miRNAs have been shown to function as either oncogenes or tumor suppressors [9]. Reduced let-7 expression was significantly associated with reduced postoperative survival [10], while the miR-17-92 cluster was reported to act as oncogenes that enhanced lung cancer cell growth [11]. Upon irradiation, multiple miRNAs are altered during the shift in the balance between radioresistance and cell death [12, 13]. For example, up-regulation of let-7, miR-210 and miR-21 induces the resistance of lung cancer cells to radiotherapy [14–16]. However, all these studies focused on a specific set of miRNAs in vitro and did not address their clinical significance.

Cells may communicate by chemical receptor-mediated events, direct cell-to-cell contact and cell-to-cell synapses [17–19]. However, attention is now being focused on cell-to-cell communication that involves spherical membrane fragments called microvesicles [20]. Exosomes are one of these membrane-derived vesicles released by many cells. They have recently been recognized as important mediators of intercellular communication, on the basis that they carry lipids, proteins, mRNAs and various microRNAs including let-7, miR-1, miR-15, miR-16, miR-181 and miR-375, which can be transferred to a recipient cell via fusion of the exosome with the target cell membrane [21]. Exosomes may play an important role in transporting radiation-induced serum miRNAs and subsequently affect the proliferation and radioresistance of human lung cancer cells.

Thus, the purpose of this study was to identify the key serum miRNAs associated with radioresistance in NSCLC. We first investigated the changes in the expression profile of the miRNAs in the serum of lung cancer patients who had been treated with radiotherapy. We then studied the role of the apparently up-regulated miRNA in the proliferation and radioresistance of NSCLC and evaluated the probable mechanism. Our findings also provide a possible explanation of the mechanisms by which NSCLC cells could acquire and regulate resistance to radiation through components of the serum.

## Methods

### Patients and serum samples

For miRNA microarray and quantitative real-time polymerase chain reaction (qRT-PCR) analyses, serum samples of lung cancer patients were obtained from Changzhou Cancer Hospital (Changzhou, China) between June 2012 and March 2014. Venous blood was drawn from 60 volunteers before radiotherapy (BR) and only half of the compliant serums were obtained 24 h after receiving 60 Gy radiotherapy (AR). All of the collected blood samples were held at room temperature for 60 min. After centrifugation at 3000 × g for 10 min, the supernatants were collected and immediately frozen at −80 °C to preserve them until further use. The patients in this study had not been subjected to chemotherapy, and their tumors were diagnosed as carcinomas on the basis of pathological evidence: the histological features of the specimens were evaluated by two senior pathologists. All patients gave signed, informed consent for their blood to be used for scientific research. Ethical approval for this study was obtained from Changzhou Cancer Hospital Affiliated to Soochow University. All experiments were performed in accordance with the relevant guidelines and regulations of Soochow University.

### Serum miRNA microarray

The miRNA microarray experiments were performed to identify the differentially expressed miRNAs. Total RNAs were extracted from the paired serum samples (before and after radiotherapy) from three patients with an age range of 54 to 66 years. All samples passed the quality control analysis, and no signs of PCR reaction inhibition were observed. A microRNA PCR panel (V3.M; Exiqon, Vedbaek, Denmark) was used to evaluate the miRNA profiles in this study. The RNAs were labeled and hybridized following the manufacturer’s instructions at KangChen Bio-tech (Shanghai, China).

### qRT-PCR validation of miRNA expression

qRT-PCR analysis was performed to evaluate the miR-208a expression in this study. The specific stem-looped qRT-PCR primers for miR-208a, miR-29b-3p, miR-126-3p and miR-200a-3p were designed by GenePharma Co. Ltd. (Shanghai, China). The qRT-PCR analysis was performed on an ABI 7500 instrument (Applied Biosystems, Foster City, CA, USA) using TaqMan-based microRNA assays according to the manufacturer’s protocols as described in our previous study [22]. U6 and miR-16 were measured by the same method and used to normalize the cell samples and serum samples, respectively. The relative quantity of each miRNA in the serum or cells, normalized to U6 or miR-16 and relative to the expression of those samples before radiotherapy, was calculated using the equation RQ = 2^-ΔΔCT^, where ΔΔCT = (CT_miRNA_ - CT_U6__or miR-16_) AR - (CT_miRNA_ - CT _U6__or miR-16_) BR, and the CT value was the threshold cycle for the detection of fluorescence. After amplification, melting curve analysis was performed to ensure the specificity of the products.

For cellular medium miRNA analysis, we first collected the medium incubated with A549 cells after 0 or 4 Gy X-ray irradiation. The supernatants were centrifuged at 7,500 × g for 40 min using an ultra-filtration membrane tube (10 kDa, Merck Millipore, Darmstadt, Germany). The concentrated solution was then harvested using TRIzol reagent (Tiangen, Beijing, China) and further for qRT-PCR analysis.

### Cell cultures and irradiation

The human NSCLC cell lines (A549, H1299, H1975 and H460) and human non-cancerous bronchial epithelial cell lines (BEAS-2B and HBE) were maintained in Dulbecco’s Modified Eagles Medium (DMEM) supplemented with 10 % fetal bovine serum (FBS) (Gibco, Grand Island, NY, USA) at 37 °C in 5 % CO_2_. The lung cancer patients received a total dose of 60 Gy X-ray from a linear accelerator (Varian, Palo Alto, CA, USA) at a dose rate of 2 Gy/min. The cells were exposed to a single dose of X-rays using a linear accelerator (RadSource, Suwanee, GA, USA) at a dose rate of 1.15 Gy/min and 160 kv X-ray energy.

### RNA synthesis and cellular transfection

The miR-208a mimics and negative control miRNA (miRNA-NC) or miR-208a inhibitor and inhibitor-NC were all synthesized by GenePharma Co. Ltd. (Shanghai, China). The synthetic RNAs were transfected into cells using Lipofect reagent (Tiangen, Beijing, China) according to the manufacturer’s instructions.

### Luciferase assays

A luciferase reporter that contained the full-length 3′UTR of p21 (pGL3-UTR-WT) and the mutant reporter plasmid with mutation of the miR-208a binding site (pGL3-UTR-MUT) were constructed by Synbio Tech, Ltd. (Suzhou, China). For luciferase assay, 500 ng of the reporter vector was cotranfected with 500 ng synthetic RNA in a 24-well plate. 30 ng of pRL-TK (Promega, Madison, WI) was also transfected to correct the transfection efficiency. The luciferase activity was measured with the Dual-Luciferase Reporter Assay System (Promega). The ratio of *Firefly* luciferase to *Renilla* luciferase activities were considered as promoter activities.

### Cell proliferation assay

The cells were seeded in a 96-well plate at a density of 2 × 10^3^ cells per well. The cells were transfected with 50 nM of either miR-208a mimics and miRNA-NC or miR-208a inhibitor and inhibitor-NC 24 h later and allowed to grow for another 48 h. The cell proliferation was measured using the 3-(4,5-dimethylthiazol-2-yl)-2,5-diphenyl-2Htetrazolium bromide (MTT) assay 48 h after the transfection with the RNAs. Briefly, 20 μL of the MTT solution (5 mg/mL) was added to each well, and the cells were incubated for another 4 h at 37 °C. The medium was then aspirated, and 150 μL of dimethylsulfoxide (DMSO) was added to dissolve the crystals. The optical density was measured at 492 nm using a microplate reader (Bio-Rad, Hercules, CA, USA). The viability index was calculated as the experimental OD value/the control OD value. Three independent experiments were performed in quadruplicate.

5-ethynyl-20-deoxyuridine (EdU) is a nucleoside analog of thymidine that is only incorporated into DNA during active DNA synthesis by proliferating cells. After incorporation, a fluorescent molecule that reacts specifically with EdU was added, which made the proliferating cells fluorescent. The Cell-Light EdU DNA cell proliferation kit (Ribo Bio., Guangzhou, China) was used to determine the proliferation rate of the A549 cells according to the manufacturer’s instructions. Briefly, the cells were incubated with 50 μM EdU for 2 h before fixation, permeabilization, and EdU staining. The cell nuclei were stained with Hoechst 33342 at a concentration of 5 μg/mL for 30 min, and the cells were examined using a florescence microscope (Olympus, Tokyo, Japan).

### Clonogenic assay

The cells (2 × 10^5^) were seeded into six-well plates and subjected to transfection the next day. The plates were irradiated with doses of 0, 2, 4, 6 or 8 Gy X-ray irradiation given in a single fraction 48 h after transfection. After incubation at 37 °C and 5 % CO_2_ for 10–14 days, the cells were subsequently fixed with methanol and stained using 1 % crystal violet in 70 % ethanol. The colonies containing 50 or more cells were counted according to our previous study [22]. SF (surviving fraction) = Number of colonies/(cells inoculated × plating efficiency). The survival curve was derived from a multi-target single-hit model: SF = 1-1-exp(-D/D_0_)^n^ [23]. D_0_ was defined as the dose that gave an average of one hit per target. The radiation sensitivity enhancement ratio (SER) was measured according to the multi-target single-hit model.

### Flow cytometric analysis of cell apoptosis and cell cycle

An annexin V/7-aminoactinomycin D (7-AAD) apoptosis kit (BD Biosciences, San Jose, CA, USA) was employed to evaluate cellular apoptosis. The cells were harvested 48 h after being transfected with the RNA and then stained with Annexin V/7-AAD for 30 min. The results were analyzed using a FACSCalibur system with ModFit’s LT software (Becton Dickinson, CA, USA). For the cell cycle analyses, 24 h after being transfected with the RNA, the cells were collected and fixed with 70 % precooled ethanol overnight. After staining with propidium iodide (10 μg/ml; Sigma-Aldrich) in the dark for 30 min, flow cytometry was performed on the FACSCalibur system, and the cell cycle distribution was analyzed using the ModFit LT software.

### Western blotting

The proteins in lysates from the cells or exosomes were resolved using sodium dodecyl sulfate (SDS)–polyacrylamide gel electrophoresis and transferred to a nitrocellulose membrane, which was then blocked with phosphate-buffered saline/Tween-20 containing 5 % non-fat milk. The membrane was incubated with antibodies to p21, AKT, p-AKT mTOR and p-mTOR (All from epitomics, Burlingame, CA, USA). The apoptotic related antibodies to PARP1, Bcl2 and Bax were all purchased from Santa Cruz Biotechnology (Santa Cruz, CA, USA). α-Tubulin (Beyotime, Nantong, China) served as the loading control. The protein-bound antibodies were detected using an enhanced chemiluminescence (ECL) Stable Peroxide solution (PointBio, Shanghai, China). All protein bands were visualized by a FluroChem MI imaging system (Alpha Innotech, Santa Clara, CA, USA) at room temperature.

### Purification and characterization of exosomes

Exosomes were prepared from the supernatants of the sera by differential centrifugation as described below. Briefly, the sera were centrifuged at 500 × g for 10 min to remove the cells and then at 16 500 × g for 20 min, followed by filtration through a 0.22 μm filter to remove cell debris. The exosomes were pelleted by ultracentrifugation (Beckman Coulter, Inc., CA, USA) at 120 000 × g for 70 min according to a previous report [21]. For qRT-PCR, Western blot assay and confocal microscopy, the pellets of the ultracentrifugation procedure were divided into three equivalent parts and examined according to the methods described above or below. Western blotting with the surface marker CD63 (Santa Cruz Biotechnology, Santa Cruz, CA, USA) was used to confirm that the extract of the pellet contained extracted exosomes [24]. The protein content of the exosomes was measured using a BCA™ Protein Assay Kit (Beyotime, Nantong, China). 40 ug of the quantified protein samples were loaded into each well of the gel in this study.

### Exosome uptake by confocal microscopy

Exosomes isolated according to the isolation protocol were washed using an ultra-filtration membrane (10 kDa, Merck Millipore, Darmstadt, Germany) to remove any free nucleotides. 1,1′-dioctadecyl-3,3,3′,3′-tetramethylindocarbocyanine perchlorate (Dil) was used to label the membrane component of the exosomes according to Thery et al. [25]. The exosomes were incubated with 10 μg/mL DiI (Beyotime, Nantong, China) for 15 min at 37 °C and then washed twice with cold phosphate buffered saline (PBS). The DiI-labeled exosomes were added to A549 cells grown on chamber slides of a 35 mm dish with sterile small circular glass and incubated at 37 °C. At the time points of 0, 30 or 90 min, the cells were harvested and washed twice with PBS. Then, the cells were counter-stained with 4’-6-diamidino-2-phenylindole (DAPI, Invitrogen, Carlsbad, CA, USA) to visualize the nuclei and were examined using an UltraView VoX confocal fluorescence microscope (PerkinElmer, Waltham, MA, USA).

### Statistical analysis

The data are expressed as the means ± standard error of the mean (SEM) of at least three independent experiments. The standard error bars are shown for all data points. A Student’s *t*-test was performed to determine the statistical significance of differences. IBM SPSS Statistical 19.0 software (IBM, NY, USA) was utilized for the statistical analyses. *P* < 0.05 was considered significant.

## Results

### Altered serum miRNA expression profiles of lung cancer patients after radiotherapy

Using miRNA microarray analysis, we evaluated the paired serum miRNA expression profiles of three lung cancer patients before and after radiotherapy. To identify the differentially expressed miRNAs on the chip, GenEx analysis was used to generate a list of differentially expressed miRNAs at FDR = 0 (0 % false discovery rate) (Additional file 1: Table S1). The significantly altered miRNAs are listed in Table 1. We identified only one miRNA (miR-208a) that was up-regulated after radiotherapy in sera of the lung cancer patients compared with the same patients’ serum before radiotherapy. Nine miRNAs were significantly down-regulated (*P* < 0.05): miR-29b-3p, miR-200a-3p, miR-126-3p, miR-26b-5p, miR-200a-3p, miR-15b-3p, let7c/7i-3p and miR-382-5p. We further validated four representative differentially expressed miRNAs (miR-208a, miR-126-3p, miR-29b-3p and miR-200a-3p) as listed in Table 1 by qRT-PCR analysis. The ratio of the PCR results (AR versus BR) was compared with miRNA microarray signal intensities to test the consistency of the two methods. Additional file 2: Figure S1 displays a comparison of qRT-PCR and microarray results for the four differentially expressed miRNAs. The qRT-PCR analysis confirmed all results obtained by microarray analysis consistently.

**Table 1:** Significant fold changes in serum miRNAs in lung cancer patients before and after radiotherapy analyzed by miRNA microarray

miRNA ID	AVG ∆C_t_		2^-∆C_t_		Fold difference		Fold up- or down- regulation	Regulation
	(Ct(GOI)-Ave Ct(HKG))							
	After RT	Before RT	After RT	Before RT	After RT/Before RT	*p* value	After RT/Before RT	
hsa-miR-29b-3p	4.929	3.756	0.033	0.074	0.444	0.002	−2.254	Down
hsa-miR-223-3p	−4.307	−4.850	19.799	28.833	0.687	0.004	−1.456	Down
hsa-miR-126-3p	−1.855	−2.132	3.618	4.382	0.826	0.008	−1.211	Down
hsa-miR-26b-Sp	–0.080	–0.436	1.057	1.353	0.781	0.025	–1.280	Down
hsa-miR-208a	13.010	14.133	0.000	0.000	2.179	0.030	2.179	Up
hsa-miR-200a-3p	9.266	7.497	0.002	0.006	0.293	0.038	–3.408	Down
hsa-miR-15b-Sp	1.646	0.935	0.319	0.523	0.611	0.039	−1.637	Down
hsa-Iet-7c	3.984	3.518	0.063	0.087	0.724	0.041	−1.382	Down
hsa-miR-382-Sp	7.302	6.578	0.006	0.010	0.605	0.042	−1.652	Down
hsa-Iet-7i-3p	8.801	8.142	0.002	0.004	0.634	0.049	−1.579	Down

### Validation of altered miR-208a expression upon radiotherapy in both lung cancer patient sera and human lung cancer cells

**Fig. 1:**
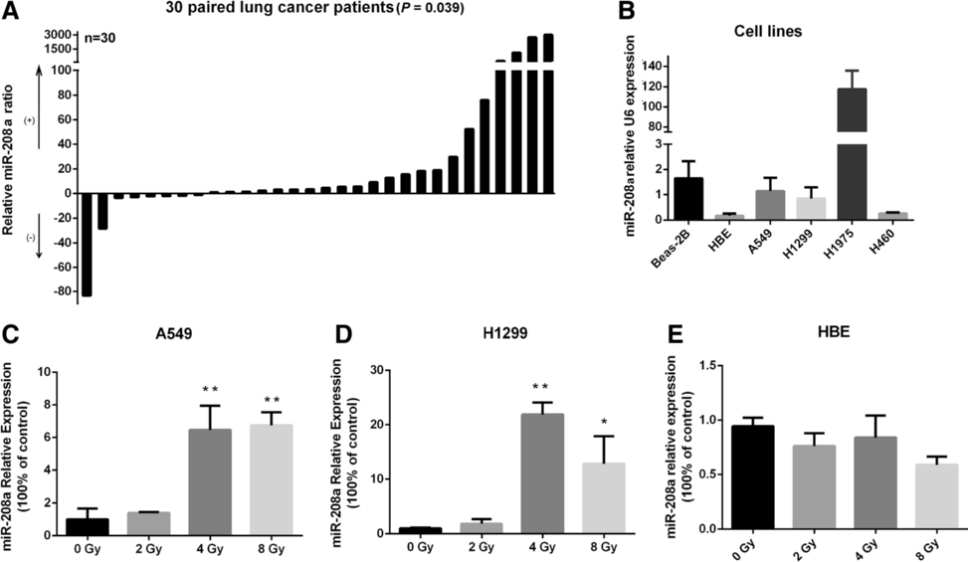
miR-208a in pairs of serum samples from 30 lung cancer patients before and after radiotherapy and in lung cancer cells was examined by qRT-PCR. **a** The average serum miR-208a expression in 30 paired serum samples from lung cancer patients before and after radiotherapy. **b** qRT-PCR analysis of the expression of miR-208a in human lung cancer cell lines (A549, H1299, H1975 and H460) and non-cancerous bronchial epithelial cell lines (BEAS-2B and HBE). The relative expression levels of miR-208a in (**c**) A549, (**d**) H1299 and (**e**) HBE cells after exposure to 0, 2, 4 or 8 Gy X-ray irradiation. The relative serum miR-208a expression level normalized to miR-16 and cellular miR-208a expression normalized to U6 were calculated using the equation described in the Materials and Methods. *P < 0.05 and **P < 0.01 compared with the negative control

Because miR-208a was the only up-regulated miRNA, we then validated its expression in 30 paired serum samples by comparing the AR samples with the corresponding BR samples. The clinical characteristics of these patients were classified and listed in Additional file 3: Table S2. As shown in Fig. 1a, miR-208a was significantly over-expressed in the AR groups compared with their corresponding BR groups with a *P* value of 0.039. Thus, for miR-208a, the qRT-PCR analysis confirmed the result obtained by microarray analysis, and the difference was even stronger (>2.178-fold) when validated in the 30 paired lung cancer serum samples than that was anticipated from the microarray studies.

We further determined the relative expression of miR-208a in the NSCLC cell lines (A549, H1299, H1975 and H460) and non-cancerous bronchial epithelial cell lines (BEAS-2B and HBE). The qRT-PCR results showed that miR-208a was expressed in both NSCLC cell lines and non-cancerous bronchial epithelial cell lines, with clearly more pronounced expression in the H1975 cells (Fig. 1b). Moreover, we found that the expression of miR-208a in A549 and H1299 cells could be further induced by X-ray irradiation (Fig. 1c and d). In contrast, miR-208a could not be induced by X-ray irradiation in the non-cancerous bronchial epithelial HBE cells (Fig. 1e). Finally, to validate the antagonistic effect in NSCLC cells, the miR-208a mimics and miR-208a inhibitor were transfected into A549 and H1299 cells. As shown in Additional file 4: Figure S2A and S2B, the mimics enhanced the miR-208a expression in the two cell lines, and the inhibitor caused a significant decrease of miR-208a expression (*P* < 0.05).

### miR-208a directly targeted p21 and affected AKT/mTOR pathway

**Fig. 2:**
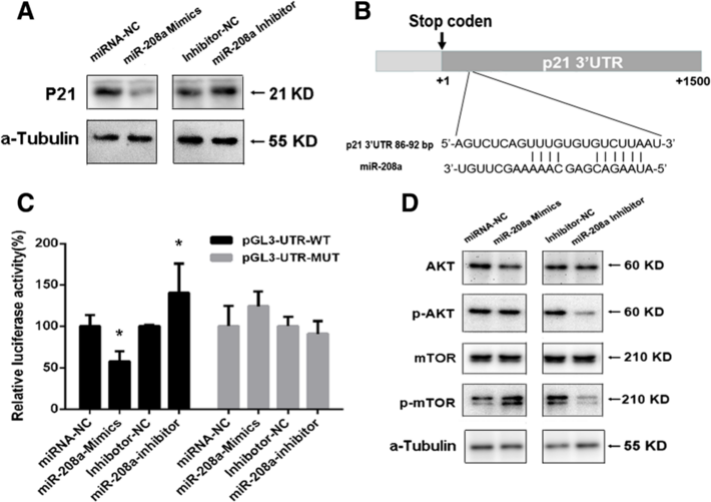
miR-208a affected the levels of the p21/AKT/mTOR pathway in A549 cells. A549 cells were transfected with the miR-208a mimics, negative control miRNA (miRNA-NC), miR-208a inhibitor or inhibitor-NC for 24 h before lysis. **a** Western blot analysis the expression levels of p21. **b** Putative miR-208a binding sequences in the 3′-UTR of p21 mRNA. **c** Full-length 3′-UTR of p21 and the mutant sequences were cloned into the downstream of the luciferase reporter gene named as pGL3-UTR-WT and pGL3-UTR-MUT, respectively. Each of the plasmids and pRL-TK, were cotransfected into A549 cells, either with miR-208a mimics or inhibitor as indicated. Luciferase activity was assayed 48 h after transfection. All experiments were repeated three times independently. The results are presented as the means ± SEM (*n* = 3). *P < 0.05. **d** The membranes were incubated with antibodies to AKT, p-AKT, mTOR, or p-mTOR. The samples were derived from the same experiment and analyzed under the same experimental conditions

To explore the mechanism by which miR-208a executes its function in lung cancer, we first analyzed its predicted target genes using three bioinformatic algorithms (TargetScan, PicTar and miRBase). Among the predicted candidates, p21 (CDKN1A) was selected for further analysis. This led us to examine whether ectopic expression of miR-208a could reduce endogenous p21 protein levels in human lung cancer cell lines. Western blot assays showed that the level of p21 protein was reduced significantly by miR-208a over-expression, whereas miR-208a down-regulation promoted p21 expression while compared with control treatment (Fig. 2a). These results indicated p21 as a target of miR-208a for its regulatory function in lung cancer.

Because the binding sites of miR-208a were predicted at positions 86-92 in the 3′-UTR of p21 mRNA (Fig. 2b). We further test the hypothesis that p21 be a target of miR-208a using luciferase assays. Reporter by putting the wild type fragments covering position 1-1500 from the 3’-UTR region of p21 to the downstream of the luciferase coding region (named pGL3-UTR-WT, Fig. 2b) was constructed. miR-208a mimics or inhibitor were each cotransfected with the above reporters into human lung cancer A549 cells. Luciferase assay showed that miR-208a mimics repressed the activity of pGL3-UTR-WT remarkably, whereas transfection inhibitor enhanced the promoter activities (Fig. 2c). In contrast, transfection of pGL3-UTR-MUT, in which the putative binding site of miR-208a was mutated, showed no interference with activity after transfection with either miR-208a mimics or inhibitor (Fig. 2c). The above results indicated that 3’UTR of p21 harbours a miR-208a binding site.

Western blot analysis confirmed that transfection with the miR-208a inhibitor decreased the levels of p-AKT and p-mTOR expression when compared with the control group. Conversely, promoting miR-208a expression increased the activity of the AKT/mTOR pathway, though it’s not significantly as the effect of the inhibitor (Fig. 2d). These results indicated that the AKT/mTOR signaling pathway is involved in the miR-208a-induced proliferation of human lung cancer cells.

### miR-208a increased human lung cancer cell growth in vitro

**Fig. 3:**
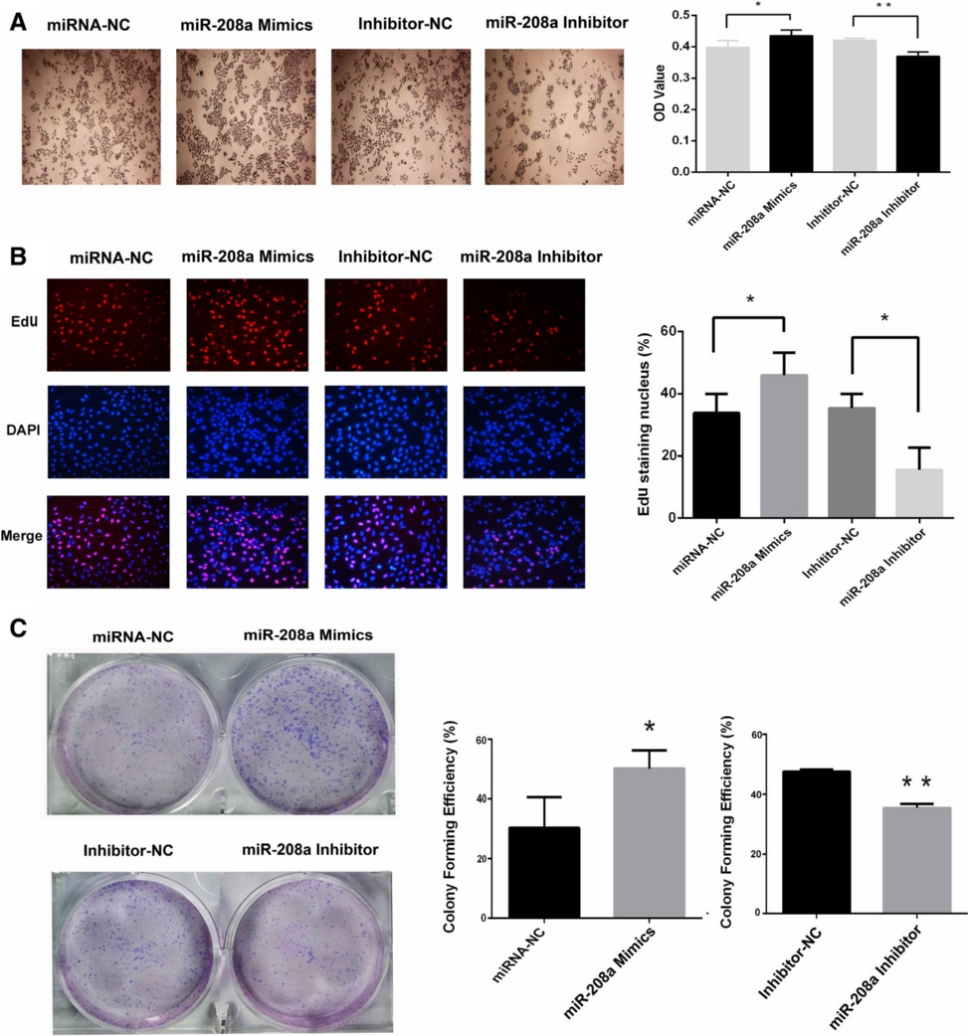
Over-expression of miR-208a promoted cellular proliferation. **a** The effect of miR-208a on viability of the A549 cells was analyzed using the MTT assay, and the miRNA-NC-transfected cells were normalized as 100 %. **b** The proliferating A549 cells were labeled with EdU. The click-it reaction revealed the EdU staining (red). The cell nuclei were stained with Hoechst 33342 (blue). The images are representative of the results obtained. **c** Colony formation assays of A549 cells transfected with miR-208a mimics or miR-208a inhibitor. One thousand cells were seeded onto each plate. After 10 days, the cells were stained with crystal violet. The colonies consisting of more than 50 cells were counted. The data are presented as the means ± SEM (*n* = 4). *P < 0.05 and **P < 0.01 compared with the negative control

The MTT assay, EdU staining and colony formation were performed to investigate the effect of miR-208a on the proliferation of A549 cells. As shown in Figs. 3a and b, over-expression of miR-208a increased the proliferation rate, and the cells transiently transfected with the miR-208a mimics exhibited higher clonogenic survival rates than cells transfected with miRNA-NC, whereas down-regulation of miR-208a caused the opposite effect (Fig. 3c, *P* < 0.05). This effect was verified by a similar result in the H1299 cells, which are also an NSCLC cell line (Additional file 5: Figure S3A and S3B). Taken together, these results showed that increased miR-208a expression promoted cell growth and colony formation.

### miR-208a decreased cellular apoptosis and disturbed the cell cycle

**Fig. 4:**
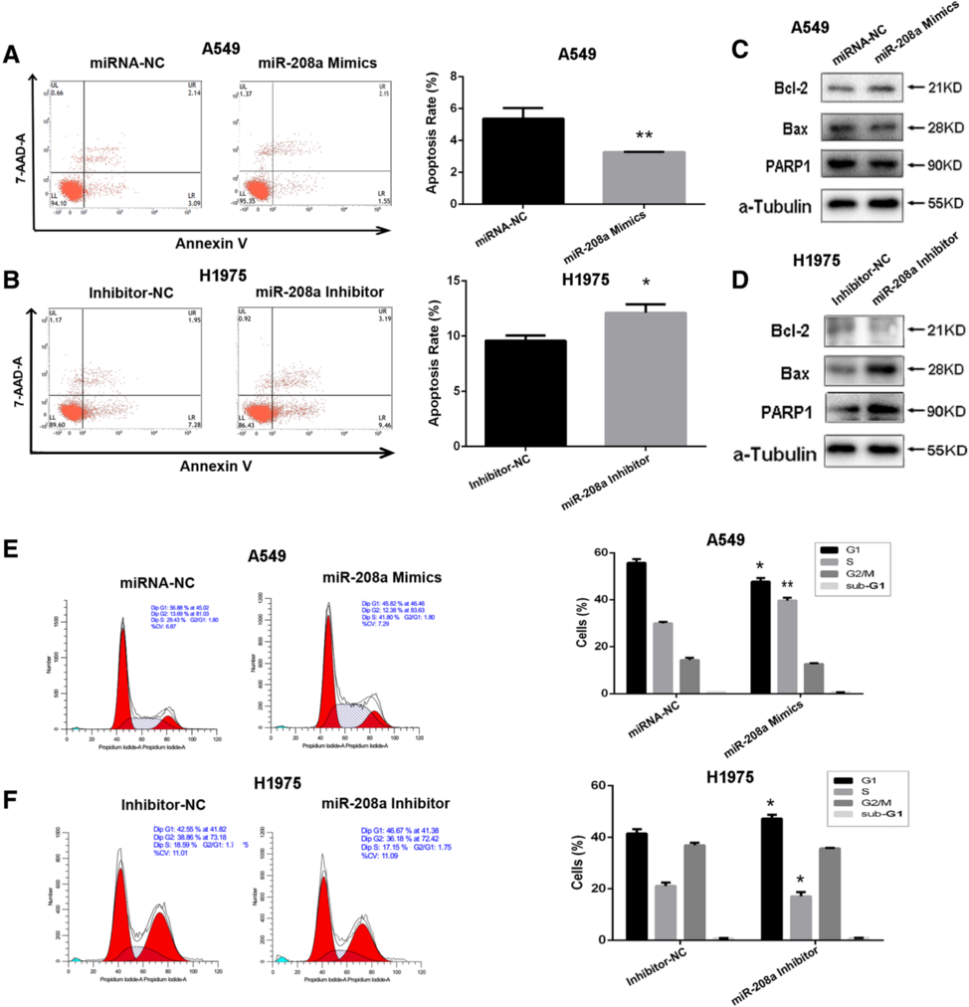
miR-208a increased the cell apoptosis and disturbed the cell cycle of lung cancer cells. **a** A549 and (**b**) H1975 cells were transfected with the indicated vectors. Two days after transfection, the percentage of apoptotic cells was evaluated using flow cytometry. Western blot analyzed the apoptotic molecular component including PARP1, Bcl2 and Bax in (**c**) A549 and (**d**) H1975 cells. The cell cycle distributions of (**e**) A549 and (**f**) H1975 cells were measured using flow cytometry. *P < 0.05 and **P < 0.01 compared with the negative control

Annexin V/7-AAD double-staining assays were conducted to analyze cellular apoptosis. The percentage of apoptotic cells decreased significantly in the A549 cells after transfection with the miR-208a mimics compared with those transfected with miRNA-NC. Specifically, the apoptosis rates were 5.28 ± 0.68 % and 3.27 ± 0.01 %, respectively (Fig. 4a). In contrast, the apoptosis rates in the miR-208a-overexpressing H1975 cells increased significantly after transfection with the miR-208a inhibitor (from 9.57 ± 0.47 % to 12.11 ± 0.76 %; Fig. 4b). We additionally addressed the apoptotic molecular component including PARP1, Bcl2 and Bax in lung cancer cells. Results showed that miR-208a mimics down-regulated the expression of apoptotic protein PARP1 and Bax and up-regulated the anti-apoptotic protein Bcl-2 in A549 cells (Fig. 4c). In H1975 cells, the apoptotic relative protein increased expression significantly after transfection with the miR-208a inhibitor (Fig. 4d), which was consistent with the results of flow cytometric analysis. However, while we further examined cell apoptosis rate after combine treated with radiotherapy, down-regulation of miR-208a enhanced apoptosis rate after receiving 4 Gy of X-ray irradiation. But transfection of miR-208a mimics did not show significant difference in our study (Additional file 6: Figure S4).

Moreover, cell cycle analysis by flow cytometry demonstrated that the percentage of cells in the S phase was significantly increased and the population of cells in the G_0_/G_1_ phase was decreased after transfection with the miR-208a mimics in A549 cells. Inhibition of miR-208a in the miR-208a-overexpressing H1975 cells produced the opposite effect (Fig. 4e and f). Taken together, these results revealed that miR-208a increased the proliferation of human lung cancer cells and decreased cellular apoptosis by modulating the cell cycle distribution in human lung cancer cells.

### miR-208a increased the radioresistance of A549 cells

**Fig. 5:**
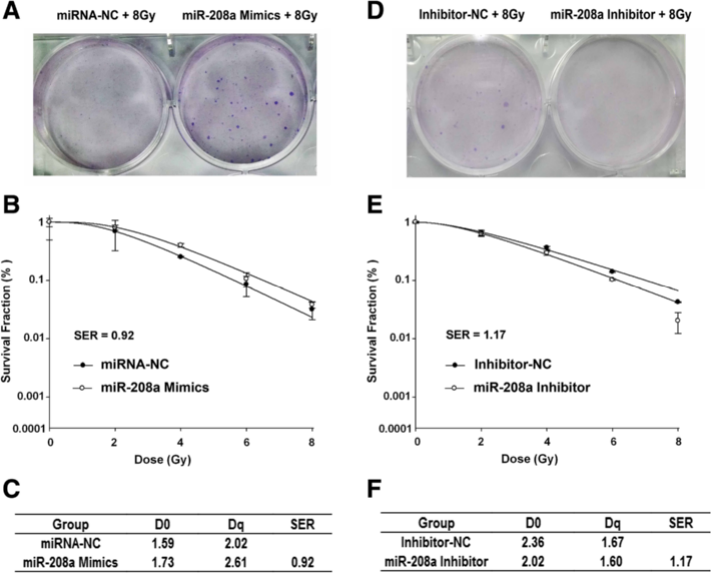
miR-208a enhanced the radioresistance of A549 cells. **a** Colony formation assays and (**b**) clonogenic cell survival curves were generated. **c** D_0_, D_q_ and calculated SER values of miRNA-NC- and miR-208a mimic-transfected groups are shown; (**d**) Colony formation assays and (**e**) clonogenic cell survival curves were generated. **f** D_0_, D_q_ and calculated SER values of inhibitor-NC- and miR-208a inhibitor-transfected groups are shown. The SER was calculated according to the multi-target single hit model

To investigate the effect of miR-208a on the radiosensitivity of human lung cancer cells, clonogenic assays were performed. Small RNAs targeting miR-208a were transfected into A549 cells followed by irradiation with a range of X-ray doses. As shown in Fig. 5a, cells transfected with miR-208a mimics before exposure to 8 Gy X-ray irradiation exhibited higher clonogenic survival rates than those treated with radiation alone. The miR-208a mimics enhanced the radioresistance of the A549 cells (SER = 0.92, Fig. 5b and c). When the miR-208a inhibitor was transfected into the A549 cells, the cells exhibited smaller clonogenic survival rates after exposure to 8 Gy X-ray rradiation and became more sensitive to irradiation (SER = 1.17, Fig. 5d, e and f). These results suggested that miR-208a enhanced the radioresistance of the A549 lung cancer cells.

### Serum exosome miR-208a was translocated into human lung cancer cells in an exosome-mediated manner

**Fig. 6:**
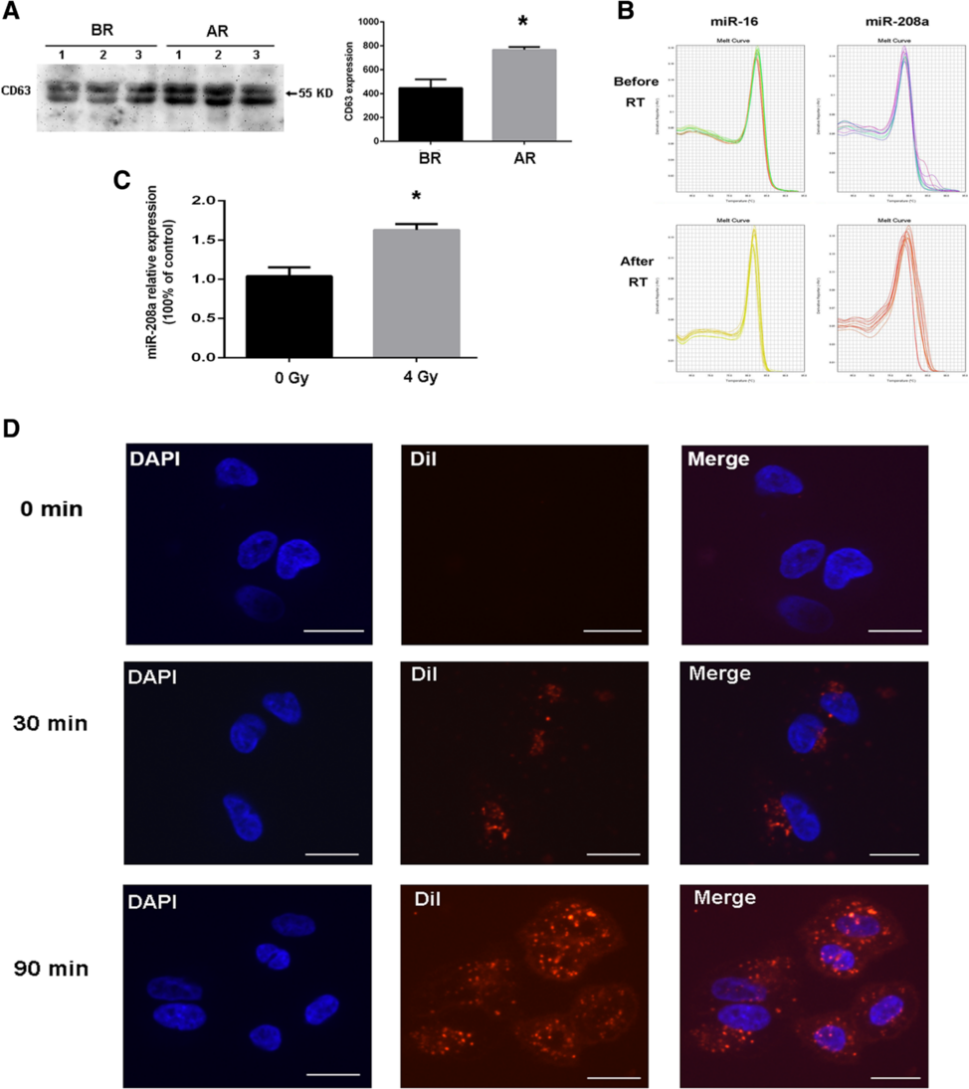
Identification and characterization of serum exosome miR-208a translocation into A549 cells. Exosomes were isolated using centrifugation, filtration and ultracentrifugation as described in the Materials and Methods. **a** Confirmation of the exosome marker CD63 by Western blot. **b** qRT-PCR analysis of miR-208a and miR-16 contained in the exosomes. **c** qRT-PCR analysis of miR-208a in culture medium of A549 cells. The conditional medium incubated with A549 cells collected 24 h after exposure to 0 or 4 Gy X-ray irradiation. **d** Microscopic analysis of the translocation of the pre-labeled exosomes. The DiI-labeled exosomes displayed red color. The cell nuclei were stained with DAPI (blue). The scale bar indicates 20 μm. *P < 0.05 compared with BR or the group treated with 0 Gy. BR: before radiotherapy; AR: after receiving 60 Gy radiotherapy

Because our study found that miR-208a was present in the serum of lung cancer patients, and exosomes have recently been recognized as important mediators of intercellular communication [21], we further investigated whether serum miR-208a can be transduced into lung cancer cells by exosomes in vitro. First, Western blot analysis with antibodies against the exosome-specific surface marker CD63 was used to confirm that the serial ultra-centrifugation of the sera resulted in purified exosomes. As shown in Fig. 6a, the CD63 expression was increased in the AR group compared with the corresponding BR group, suggesting that exosomes can also be induced by radiotherapy in the serum. Then, the qRT-PCR results showed that miRNAs including miR-208a as well as the ubiquitous miR-16 were present in the purified exosomes (Fig. 6b). To illustrate the major source of miR-208a after radiotherapy, we detected miR-208a expression in the culture medium of A549 cells after 0 or 4Gy X-ray irradiation by qRT-PCR analysis. Results showed that radiotherapy can induce miR-208a secretion from A549 cells to the culture medium (Fig. 6c). We concluded that the source of radiation induced miR-208a was secreted from lung cancer cells.

Next, exosomes containing miRNAs were labeled with the membrane dye DiI. The labeled exosomes were added to the culture medium of the A549 cells. After co-culture for 0, 30 or 90 min with unlabeled recipient cells, the labeled exosomes as well as A549 cells were observed using a confocal microscope. The results showed that the exosomes containing miR-208a could be incorporated into A549 cells in a time-dependent manner. Our study demonstrated that serum exosomes containing miRNAs can be taken up in turn by lung cancer cells (Fig. 6d) and may subsequently mediate cellular functions.

## Discussion

Radiation exposure increases the levels of intracellular free radical species, followed by DNA strand breaks and subsequent dysfunction of the mitochondria, endoplasmic reticulum and other organelles [26, 27]. These radiation-induced cellular events lead to activation of pro-apoptotic signaling and eventually to tumor cell killing [28]. Several studies on the mechanisms of radioresistance in NSCLC have suggested the potential involvement of either p53 mutations, altered expression of survival proteins including X-linked inhibitor of apoptosis protein (XIAP) and survivin, or activation of phosphoinositide 3-kinase (PI3K)/AKT signaling [29]. Identification of molecular factors responsible for conferring radioresistance and developing new strategies that increase sensitivity to radiation is warranted.

Accumulating evidence has implicated miRNAs in the radiosensitivity of various types of cancer cells. Transient over-expression of miR-181a confers resistance of cervical cancer cells to radiation therapy by targeting the pro-apoptotic PRKCD gene [23]. High levels of serum miR-155 and miR-221 are associated with the development of severe radiation-induced esophageal toxicity during an early stage of radiation therapy for non-small cell lung cancer [30]. However, high-throughput analysis of the serum miRNA expression profile associated with the radiosensitivity of human lung cancer cells has not been reported. Our study demonstrated that radiotherapy produces multiple alterations in the miRNA expression profile in the serum of lung cancer patients and that miR-208a was notably increased in the serum after exposure to a total of 60 Gy X-ray irradiation. Interestingly, our study showed that the expression of miR-208a could be induced by X-ray irradiation in lung cancer A549 and H1299 cells but not in the non-cancerous bronchial epithelial HBE cells, suggesting that lung cancer cells were likely to be the major source of increased miR-208a after radiotherapy. We also found that irradiation increased miR-208a in the culture medium of A549 cells, which further supported this hypothesis. Subsequently, we focused our study on the biological function of the serum miR-208a and found that increased miR-208a played a role in a negative-feedback mechanism that promoted the proliferation and radioresistance of lung cancer cells.

Studies have shown that miR-208 induces the epithelial to mesenchymal transition of pancreatic cancer cells and thereby promotes cell metastasis and invasion [31]. Alternatively, another recent report suggested that miR-208 is a potential onco-miRNA and participates in the carcinogenesis of esophageal squamous cells by suppressing SOX6 expression [32]. miR-208a, one of the mature products of miR-208, which is enriched in the heart, plays a crucial role in heart health and disease. Previous studies have identified miR-208a as a potential therapeutic target for systemic metabolic disorders that affect the heart [33, 34]. This miRNA was reported to increase endoglin expression to induce myocardial fibrosis with acute myocardial infarction (AMI) [35]. Our study expands the list of diverse functions of miR-208a in driving lung cancer proliferation and radiation response. However, whether miR-208a could serve as a potential diagnostic and therapeutic target in cancer merits needs further multi-central clinical studies.

p21, a p53-inducible protein, is a critical regulator of cell survival and cell cycle by inhibiting both the DNA synthesis regulator proliferating cell nuclear antigen and activation of cyclinD1-CDK4/6 complexes [36, 37]. Furthermore, elevated p21 protein levels have been observed in human colon cancer as well as linked to radioresistance [38]. PI3K/AKT/mTOR pathway activation plays important roles in NSCLC via both apoptosis and autophagy inhibition pathway [39]. mTOR is commonly activated in NSCLC and is considered to be a potential target for the treatment of NSCLC patients [40]. p21-activated kinase can directly phosphorylate Akt at Ser473, suggesting that p21 may be a relevant PDK2 responsible for AKT phosphorylation [41]. In our study, we identified p21 is directly targeted by miR-208a, and correspondingly activated AKT and modulated its downstream effector mTOR in A549 cells, which was consistent with the results of Chen, et al. [42]. These results suggested that the p21/AKT/mTOR pathway was involved in the ability of miR-208a to increase the proliferation of human lung cancer cells. Our report also showed that up-regulation of miR-208a decreased the percentage of cells undergoing apoptosis and promoted G1 phase arrest, while inhibition of miR-208a in H1975 cells, which over-expressed this miRNA, results in the opposite effects. These results are consistent with the theory that disturbing the balance between proliferation and cell death by disruption of the program that regulates cell cycle entry and death can result in cellular carcinogenesis [43].

Exosomes have been reported to be minimally invasive, novel and sensitive biomarkers for prospective tracking and early detection of tumor recurrence [44, 45]. Exosomes can mediate communication between hepatocytes and monocytes/macrophages [24]. Hepatocyte-derived miR-122 can reprogram monocytes and induce sensitization to lipopolysaccharide [24]. Exosomal miRNAs are promising for therapeutic applications, not only for cancer therapy but also for regenerative medicine [46]. Our results showed that the exosomes isolated from the sera of lung cancer patients before or after radiotherapy contained miRNA-208a and were taken up by the recipient lung cancer cells. Thus, our study provides proof-of-concept that exosomes can participate in a novel and intricate process by which lung cancer cells communicate and may serve as a proliferation-stimulating and radioresistance-inducing signal by which the cells may interact with each other. However, there is still much to be learned regarding the processes by which exosomes are involved in cellular signal transduction. One recent report showed that miRNA sorting to exosomes was modulated by cell activation-dependent changes in the miRNA target levels in the producer cells [47]. If exosomes interact with a recipient cell through specific receptor–ligand interactions, whether this venue of communication could be expected to be cell type-specific and the mechanisms whereby miRNAs are sorted to exosomes demand further research.

## Conclusion

In conclusion, we evaluated the alterations in the serum miRNA profiles of lung cancer patients after radiotherapy. Among the differentially expressed miRNAs, miR-208a promoted lung cancer cell proliferation and decreased cell apoptosis by targeting p21 and AKT/mTOR pathway. Our demonstration that miR-208a is present in exosomes supported the hypothesis that exosomes may be a vehicle by which to modulate recipient cell functions. Our findings suggest that miR-208a served as a novel tumor accelerator miRNA and may be a potential therapeutic target for the treatment of human lung cancer.

## Additional files

Additional file 1: Table S1. Fold changes of all miRNAs in lung cancer after radiotherapy analyzed by miRNA microarray (sorted by *P* value from small to large). (XLS 126 kb) Additional file 2: Figure S1. Comparison of miRNA fold changes between miRNA microarray and qRT-PCR. The fold change of differentially expressed miRNAs (miR-29b-3p, miR-126-3p, miR-208a and miR-200a-3p) was determined for lung cancer patients after radiotherapy (AR) in comparison with before radiotherapy (BR). Microarray fold change was calculated by the ratio of miRNA signal intensities in AR versus BR. qRT-PCR fold change is the PCR value (AR versus BR, see Materials and Methods). (TIF 41 kb) Additional file 3: Table S2. Clinical characteristic of the lung cancer patients. (TIF 41 kb) Additional file 4: Figure S2. qRT-PCR validated the antagonistic effect of miR-208a mimics and miR-208a inhibitor in NSCLC cells. Relative miR-208a expression in A549 (A) and H1299 (B) cells following transfection with miR-208a mimics or inhibitors. miR-208a expression was normalized to U6 expression. **P* < 0.05 and ***P* < 0.01 compared with the negative control. (TIF 86 kb) Additional file 5: Figure S3. Over-expression of miR-208a promoted H1299 cell proliferation. (A) Proliferating H1299 cells were labeled with EdU. The click-it reaction revealed EdU staining (red). The cell nuclei were stained with Hoechst 33342 (blue). The images are representative of the results obtained. (B) Colony formation assays of H1299 cell transfected with miR-208a mimics and the miR-208a inhibitor. One thousand cells were seeded onto each plate. After 10 days, the cells were stained with crystal violet. The colonies consisting of more than 50 cells were counted. The data are presented as the means ± SEM (*n* = 4). **P* < 0.05 and ***P* < 0.01 compared with the negative control. (JPG 1411 kb) Additional file 6: Figure S4. miR-208a modulated radiation-induced cell apoptosis. 24 h after tranfection of miR-208a mimics or inhibitor, A549 cells were exposed to 0 or 4 Gy X-ray irradiation. Cells were then harvested for apoptosis analysis using flow cytometry. Data are expressed as means ± SEM from three separate experiments. ***P* < 0.01 compared with the specified negative control. (TIF 50 kb)
