# Erratum to: Radiation-induced miR-208a increases the proliferation and radioresistance by targeting p21 in human lung cancer cells

**DOI:** 10.1186/s13046-016-0299-x

**Published:** 2016-01-28

**Authors:** Yiting Tang, Yayun Cui, Zengpeng Li, Zhuqing Jiao, Yong Zhang, Yan He, Guangxia Chen, Qunyan Zhou, Wenjie Wang, Xifa Zhou, Judong Luo, Shuyu Zhang

**Affiliations:** Department of Radiation Oncology, Changzhou Cancer Hospital, Soochow University, Changzhou, 213001 China; Department of Radiation Oncology, Anhui Provincial Hospital, Hefei, 213001 China; State Key Laboratory Breeding Base of Marine Genetic Resources, Third Institute of Oceanography, State Oceanic Administration, Xiamen, 361005 China; Department School of Information Science and Engineering, Changzhou University, Changzhou, 213164 China; Department of Radiation Oncology, Shandong Cancer Hospital and Institute, Shandong University, Jinan, 250117 China; School of Radiation Medicine and Protection and Collaborative Innovation Center of Radiation Medicine of Jiangsu Higher Education Institutions, Soochow University, Suzhou, 215123 China; Department of Gastroenterology, First People’s Hospital of Xuzhou, Xuzhou, 221002 China; Department of Gastroenterology, Wuxi People’s Hospital Affiliated to Nanjing Medical University, Wuxi, 214002 China

Unfortunately, the original version of this article [1] contained an error. The name of one of the authors was included incorrectly and it read “Guangxia Cheng” instead of “Guangxia Chen”.

The name has been updated in the original article and is also correctly included in full in this erratum.
