# Supplementary figures and images for: Radiation-induced miR-208a increases the proliferation and radioresistance by targeting p21 in human lung cancer cells
Source: J Exp Clin Cancer Res. 2016 Jan 12;35:7. doi: 10.1186/s13046-016-0285-3 (PMC4710038; doi:10.1186/s13046-016-0285-3)

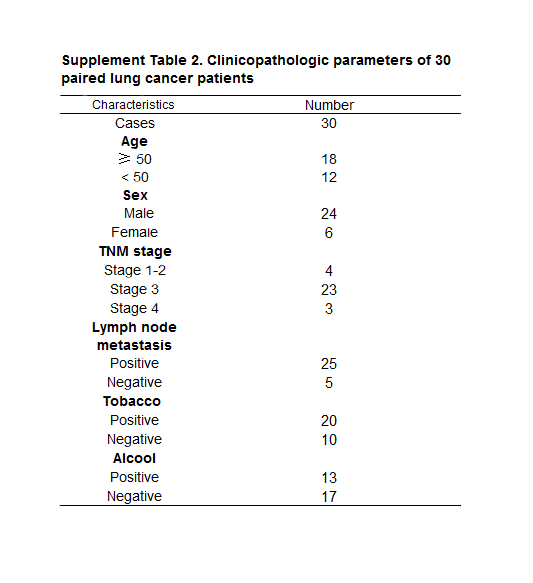

Supplement: Additional file 3: Table S2. — Clinical characteristic of the lung cancer patients. (TIF 41 kb) [file 13046_2016_285_MOESM3_ESM.tif]

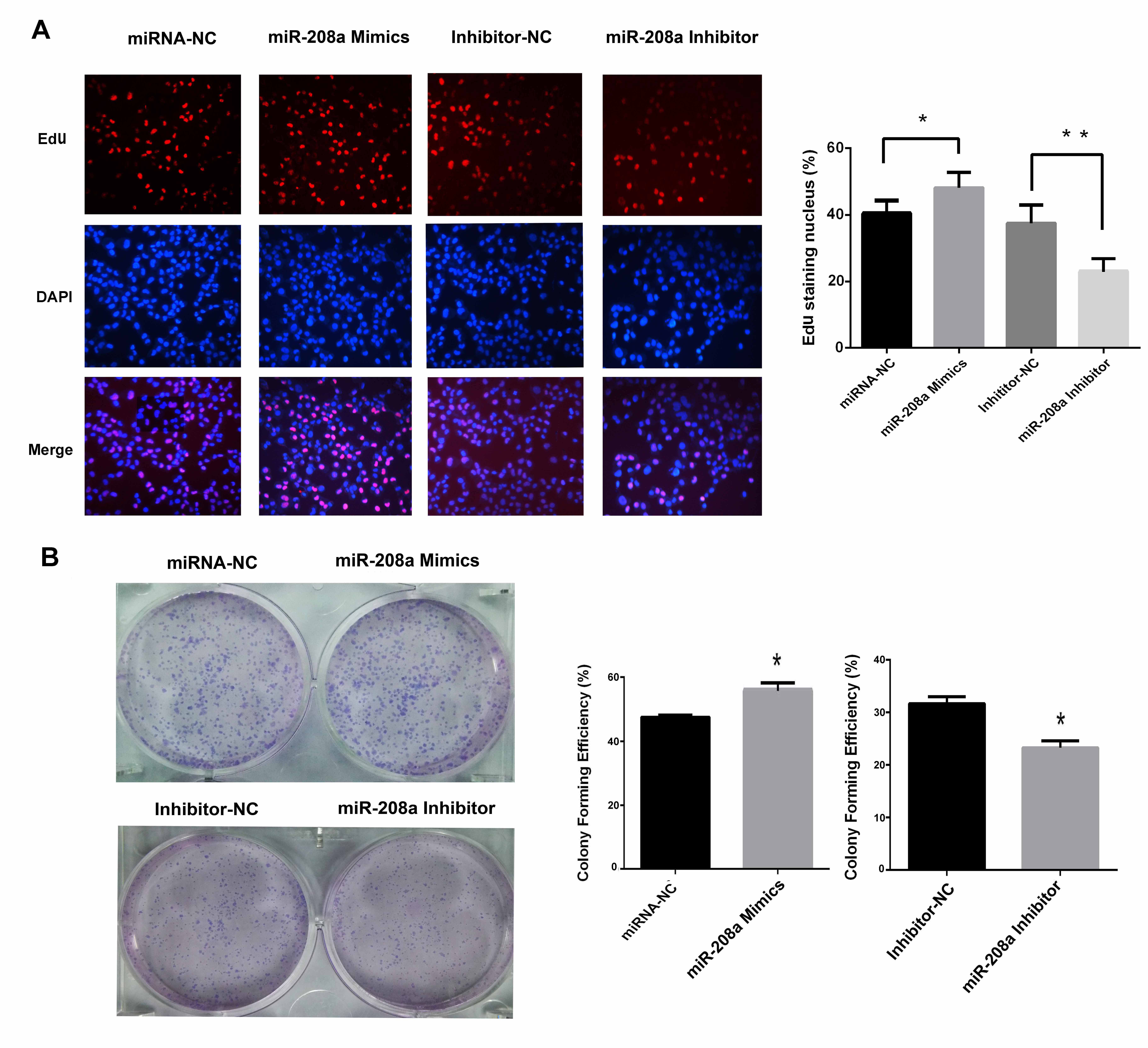

Supplement: Additional file 5: Figure S3. — Over-expression of miR-208a promoted H1299 cell proliferation. (A) Proliferating H1299 cells were labeled with EdU. The click-it reaction revealed EdU staining (red). The cell nuclei were stained with Hoechst 33342 (blue). The images are representative of the results obtained. (B) Colony formation assays of H1299 cell transfected with miR-208a mimics and the miR-208a inhibitor. One thousand cells were seeded onto each plate. After 10 days, the cells were stained with crystal violet. The colonies consisting of more than 50 cells were counted. The data are presented as the means ± SEM (n = 4). *P < 0.05 and **P < 0.01 compared with the negative control. (JPG 1411 kb) [file 13046_2016_285_MOESM5_ESM.jpg]
